# Commentary: Loss of Function Dysbiosis Associated with Antibiotics and High Fat, High Sugar Diet

**Published:** 2019-05-17

**Authors:** Aaron W. Miller

**Affiliations:** 1 Department of Urology, Cleveland Clinic, Cleveland, OH, USA; 2 Department of Cardiovascular and Metabolic Sciences, Cleveland, OH, USA

**Keywords:** Dysbiosis, Antibiotics, High fat, High sugar

Over the last decade, research into the microbiome has blossomed due in part to advances in high throughput analytical technologies (collectively considered “multi-omics”) that allow for the inquiry of highly complex biological systems such as the host-associated microbiome^1^. During this time, considerable evidence has emerged for the important role that the microbiome plays in maintaining health^2,3^. The importance of the microbiome in health and disease is derived in part from the fact that the number of bacteria that inhabits our bodies is roughly equal to the number of our own human cells and that the number of microbial genes present in and on our bodies outnumbers our own genes by a factor of 20–30^4,5^. Thus, while most of the attention on the microbiome has historically focused on infectious disease, given recent evidence of the ubiquity and health benefits of the microbiome, infectious disease should be considered the exception rather than the rule.

With the discovery of antibiotics nearly 100 years ago, we experienced a cultural shift that changed the medical field and made previously lethal infections manageable^1^. Additionally, the industrial revolution brought about agricultural advances that led to both an increase in per capita caloric intake and a shift in the sources of those calories^2^. While the advent of antibiotics and a shift towards greater availability of high caloric foods has considerably reduced deaths due to infectious disease and malnutrition, these cultural trends coincide both spatially and temporally with the emergence of non-communicable, chronic diseases such as obesity, diabetes, allergies, and other inflammatory disorders^3^. The spatiotemporal link between antibiotic use, changes in diet, and the rise of numerous chronic diseases led to the hypothesis that changes to the microbiome was driving these disease processes^4,5^. However, testing this hypothesis has been difficult, due in part to a lack of an underlying framework to determine cause and effect for such a complex system^6^. One way to determine causality between microbiome and disease states has been to perform microbial transplants into germ-free mice. Such studies have been conducted for obesity, liver disease, and encephalomyelitis among other disease states^7–9^.

Many clinical microbiome studies have been microbiome-wide association studies (MWAS)^10^. In these studies, authors will typically link a difference in microbiome composition between a healthy and disease population as ‘dysbiosis’^11^. However, two relatively unique aspects of the microbiome make this definition of dysbiosis problematic. First, given the high levels of diversity within the microbiome, the data from the high-throughput studies often used to define dysbiosis are necessarily hypervariable. Second, there is considerable inter-individual and temporal variability in microbiome composition^12,13^. Taken together, this means that when you examine two populations with completely stochastic metadata and no distinguishing phenotypes to define the populations, there will be significant differences in at least some components of the microbiome, even though no common form of dysbiosis exists within any one of the populations. Thus, a more concrete definition of dysbiosis is needed.

In Miller *et al*. 2019^14^, we specifically defined two different types of dysbiosis. Gain of function dysbiosis is that associated with infectious disease, whereby a shift in the host microbiome leads to the emergence of pathogenic bacteria and associated functions that then give rise to disease processes. Loss of function dysbiosis is defined by a shift in the host microbiome whereby bacteria that normally provided beneficial functions are lost and disease processes arise as a result. Additionally, a combination of loss and gain of function dysbiosis may be required for disease. Such is the case of chronic infection by *Clostridioides difficile,* whereby chronic antibiotic use can lead to the eventual overgrowth by pathogenic *C. difficile*^15^.

To move beyond simple MWAS studies and determine if the microbiome actually contributes to a disease or disease process is relatively straightforward. If antibiotics or some other bactericidal factor, such as those from dietary sources^16^, eliminates a disease or disease process or if microbial transplants cause a disease or disease process, then gain of function dysbiosis contributes to the disease. If the bactericidal factor leads to the emergence of a disease or disease process, or microbial transplants eliminate the disease or disease process, then loss of function dysbiosis contributes to the disease. Finally, if neither bactericidal treatments nor microbial transplants impact disease processes, then microbial dysbiosis does not contribute in any way.

In Miller *et al*.^14^, we focused on a specific microbial function, oxalate metabolism, that is exclusive to bacteria in the gut. Specific oxalate-degrading bacteria have been negatively correlated to both antibiotic use and urinary stone disease (USD) as determined by PCR or culture-based means and suggests that these bacteria are important for the inhibition of USD^17,18^. For cause-effect studies of dysbiosis, it is important to identify the specific microbial functions associated with disease in order to determine if changes in the microbiome actually causes disease or is the result of the disease. Thus, in Miller *et al.*^14^, we confirmed in animal studies that both antibiotics (Cefazolin) and a high fat, high sugar diet leads to a persistent loss of oxalate-degrading bacteria and their function. Interestingly, oxalate metabolism did not return in our antibiotic-treated animals after nine days, even though much of the gut microbiota composition did, which suggests that necessary microbe-microbe interactions were not restored.

Once it has been determined that dysbiosis does contribute to a disease or disease process, then it is important to further determine the specific location where dysbiosis is most important for the disease. Given the density and diversity of microbes in the gut, many studies focus solely on dysbiosis associated with the gut microbiota, giving rise to terms such as the ‘gut-brain axis’, ‘gut-liver axis’, or ‘gut-kidney axis’. However, in a recent clinical study, we found that even though patients with USD exhibited dysbiosis associated with the loss of the oxalate-degrading microbial network in the gut, all quantified metrics including association with antibiotic use, integration with metabolomic data, and association with other disease risk factors pointed to the urinary tract microbiome as having a greater level of dysbiosis than the gut microbiome^18^. Oral antibiotics can drive the loss of microbial diversity at many sites and lead to dysbiosis. Thus, for the development of bacteriotherapy designed to restore microbial functions, it is important to understand what forms of dysbiosis are most relevant for a disease.

As the biomedical microbiome field matures, it is important to move towards a mechanistic understanding of dysbiosis. This understanding can be achieved through a systematic process that asks the questions: 1) Does dysbiosis contribute to the disease?; 2) What locality of dysbiosis is most relevant to disease processes?; and 3) What microbial functions are lost or gained that contributes to the emergence of disease? Beyond MWAS studies, these questions must be addressed through a combination of bactericidal treatments/associations and microbial transplants of whole communities. However, to refine the specific microbial mechanisms associated with disease, specifically defined consortia of bacteria must be used for transplants ideally in combination with genetic knock-outs or knock-ins. It is only through these types of studies that the microbiome can be definitively linked to specific disease processes.
